# Connections Between Daily Greenness Exposure and Health Outcomes

**DOI:** 10.3390/ijerph17113965

**Published:** 2020-06-03

**Authors:** Xiangrong Jiang, Linda Larsen, William Sullivan

**Affiliations:** Department of Landscape Architecture, University of Illinois at Urbana-Champaign, Champaign, IL 61820, USA; lflarsen@illinois.edu (L.L.); wcsulliv@illinois.edu (W.S.)

**Keywords:** daily exposure to nature, urban nature, tree canopy, understory vegetation, street view images

## Abstract

A compelling body of research demonstrates that exposure to nature, especially trees, is beneficial to human health. We know little, however, about the extent to which understory vegetation that does not reach the height of trees, impacts human health. An additional gap in our knowledge concerns the extent to which daily variations in exposure to various forms of vegetation are related to human health outcomes. Many previous findings describing such connections were achieved in laboratory settings or through semi-controlled experiments, which do not reflect the dynamic variations of people’s daily exposure to nature. Thus, we conducted an online survey to address these questions. We used the National Land Cover Dataset 2011 and Google Street View images to estimate participants’ daily exposure to nature, and two standard questionnaires (General Health SF-12 and the Perceived Stress Scale) to assess health. Results show that greater exposure to trees in daily life is associated with better health outcomes. Specifically, higher neighborhood concentrations of tree canopy are related to better physical health, overall health and an increased capacity to control stress. In contrast, the results exploring the health associations of understory vegetation were inconsistent. In most cases, understory vegetation had a negative relationship with stress and mental health measures.

## 1. Introduction

The environments in which we live, work, and play impact our ability to face the challenges of a competitive, complex, information-rich world. These places also impact our health. The environments people are exposed to on daily basis influence their physical and mental health as well as their social cohesion [1,2,3]. Understanding the health benefits from daily exposure to built environments can help us make evidence-based decisions in support of urban forestry, urban planning, and urban design.

Although we have accumulated considerable empirical evidence regarding urban nature’s contribution to health, we do not know the extent to which daily variations in exposure to nature are related to health outcomes. There are three challenges to overcome in closing this gap in our understanding. The first challenge is that it is difficult to quantify exposure to nature in people’s daily lives. Previous studies have examined variations in nature density around people’s homes or schools as an indicator of exposure to greenness [4,5,6]. People, however, spend time at multiple geographic locations most days and the areas in which people conduct their daily routines also affect health outcomes [7]. Researchers have also realized that the amount of time spent in green spaces is a weak but significant mediator for the relationship between nature exposure and health [8]. Recently, scholars have employed a time-weighted average method to calculate vegetation density [9]. The vegetation density of places where people spend more time has greater weight than the vegetation density of places where people spend less time. Thus, the spatiotemporal dynamics of an individual’s daily nature exposure should be incorporated in our measurements of exposure to nature.

The second challenge concerns how we might quantify the concentration of different types of nature (e.g., tree canopy and understory vegetation) to which people are exposed. Most studies reporting connections between exposure to nature and human health use either the Normalized Difference Vegetation Index (NDVI), a measure derived from satellite data [10,11] or the density of tree canopy [12,13] as the measure of nature. With the growing emphasis on understory vegetation in urban landscapes [14,15], there is a clear need to understand the extent to which understory vegetation impacts human health. Understanding how various forms of vegetation are associated with human health is important if we want to make informed decisions in urban design and planning.

The third challenge is to understand how variations in measuring vegetation are related to health outcomes. There is reason to believe that the strength of the relationship between nature exposure and health outcomes may depend on whether we measure the density of vegetation from overhead images or from Google Street View images [16]. The vast majority of studies have employed satellite imagery and aerial images to calculate vegetation. But people perceive their living environment dynamically at eye-level. Street view images at eye-level may be a better way to represent and measure the environments people encounter.

Thus, we need a more inclusive measure of the nature people experience on a daily basis—around their homes, at their main daily destinations, and along their travel routes. We also need a more comprehensive method to quantify exposure to nature that includes overall vegetation density, density of tree canopy, and density of understory vegetation. Finally, we should take time into consideration and weight nature exposure by the amount of time individuals spend in various places.

In addressing these challenges, we begin by reviewing recent studies that examine exposure to nature and human health outcomes and summarize various ways to quantify exposure to nature in people’s daily lives. Next, we report an online survey of people’s daily routines and health status that examines the relationship between daily exposure to nature and health outcomes. We conclude by discussing the implications of the findings for scholars and practitioners.

## 2. Nature and Health

Exposure to nature enhances the resources necessary to manage the demands and pressures of modern life. Many people appreciate walking in the woods, watching flowing rivers, and listening to birds sing. People look for places that can refresh their mind and that encourage them to exercise and strengthen their social connections. There is abundant evidence suggesting that nature can promote both mental and physical health. We have summarized and categorized these benefits below.

Exposure to nature contributes to physical health in a variety of ways. Compared to individuals whose exposure to nature was low, those with greater exposure had healthier base-level blood pressure [17], reduced potential risks of cardiovascular disease [18], lower cortisol levels [19] and better sleep quality and quantity [20]. All of these factors are indicators that can directly predict people’s physical health.

Exposure to nature is also beneficial for mental health. Viewing or walking in green spaces can improve people’s short-term memory and their ability to concentrate [1,13,21,22,23]. In Chicago public housing, girls with greener window views had better self-discipline [24] and adults participated in less self-reported aggressive and violent behavior [25]. Exposure to nature is also linked with a lower risk of depression [26] and mood disorders [27]. Moreover, settings that include trees and open green spaces have been shown to aid stress recovery [19,28]. Finally, access to vegetation contributes to a better workplace attitude and lower level of stress [29].

Researchers have accumulated a good deal of evidence regarding nature’s impact on human health. But the understanding of the impact of nature on health is almost entirely based on measurements of nature that focus on trees that exist in limited geographic settings such as a neighborhood or a schoolyard. The nature we experience in daily life includes more diverse forms of nature and is frequently not limited by narrow geographic boundaries. To what extent does exposure to various kinds of vegetation people experience in their daily lives predict health outcomes?

## 3. Measuring People’s Daily Exposure to Nature and Health Outcomes

There are many challenges to making comprehensive measurements of people’s daily exposure to nature. In this section, we review the literature and summarize the current state of our knowledge.

### 3.1. The Spatiotemporal Dynamics of Nature Exposure

Measuring variations in exposure to nature is not an easy task [16]. One major challenge researchers face is how to quantify people’s exposure to nature in a dynamic context. In previous studies, researchers located people’s homes or schools and created a buffer around those places. The vegetation density within a buffer would represents people’s exposure to nature. Studies have explored the relationship between vegetation density around homes or schools and health outcomes. In a prospective cohort study assessing risk factors for chronic disease among women, higher levels of vegetation were associated with decreased mortality in the United States [30]. Similar results have been reported in China. People living in areas with the highest quantile of vegetation density had 27% to 30% lower mortality than those living in the lowest quantile [31]. Health inequalities related to income deprivation in all-cause mortality and mortality from circulatory diseases were lower in populations living in the greenest areas in England [32]. Vegetation density around schools predicts students’ academic achievement. For instance, tree cover density predicted students’ math achievement in Chicago public schools [14]. It was also positively associated with academic performance in public high schools in Illinois [4].

In the studies above, buffers with various radii were created around places of interest. But the selection of buffer sizes is somewhat arbitrary: radii of buffers often range from a quarter mile to several miles, depending on the resolution of data and research questions. Smaller buffer sizes are used (e.g., 0.25 or 0.5 mile) when other measures of greenness density are also employed (e.g., Google Street View Images) [31]. Still, longer radii (2 or 3 miles) have been employed when the overall vegetation density within a certain area is the main measurement [4].

To calculate vegetation density within these buffers, many previous studies have employed a top-down perspective to measure the amount of greenness within a buffer area by LiDAR [14], land cover characteristics, such as Generalized Land Use Database (GLUD) in England [33] and National Land Cover Database (NLCD) in the United States [34]. Researchers have employed Green Space of GLUD, classified-aerial images, and Tree Canopy of NLCD to quantify the concentration of nature [4,32,35].

Overall, vegetation density within an area can represent people’s exposure to nature, but the density of the vegetation tells us nothing of the temporal dynamics of people’s daily life. The temporal dynamics of an individual’s daily exposure to vegetation is another factor that should be incorporated in the estimation of their contact with nature. Recently, a time-weighted average method of calculating nature exposure has been employed to calculate the vegetation density in places where people have been [9]. The vegetation density of places where people spend more time has greater weight than those where people spend less time.

Researchers have realized that time spent in green spaces is a weak but significant mediator for the relationship between nature exposure and health [8]. For instance, people who live in greener neighborhoods but work in less green contexts tend to report worse health [7]. Moreover, in a longitudinal study in UK, researchers found that people who moved to greener urban areas had sustained mental health improvements [36]. For those living in areas of moderate greenness, increasing greenness was associated with better mental health for men older than 30 years and women older than 41 years [37]. People have lower mental distress and higher well-being when living in areas with more green spaces after controlling for individual and regional covariates [38].

Still, time of exposure to green spaces does not always predict health outcomes. In a study in Holland, although researchers found significant associations between Euclidean distances to the nearest green space and mental health outcomes in cross-sectional analysis, there was no evidence of associations between changes in green spaces and changes in mental health in the longitudinal analysis [39]. The inconsistent results suggest further research is needed to provide more evidence regarding exposure to nature and health benefits. The main limitation is the limited number of studies and the heterogeneity regarding nature exposure assessment [40].

To address the gap in our knowledge, we have developed a variable of nature exposure that combines vegetation density at multiple geolocations and exposure durations. That is, we have explicitly measured the relationship between the density of vegetation and the amount of time people spend exposed to that vegetation. With a more comprehensive estimation of people’s spatiotemporal dynamics of nature exposure, we can assess the extent to which variations in daily exposure to vegetation (e.g., in neighborhoods, daily destinations, and routes) predict human health.

### 3.2. Impacts of Different Types and Perspectives of Nature on Nature Exposure Measures

In previous studies, researchers have employed a top-down perspective to quantify the amount of greenness within a buffer using aerial photos or other land cover data. The density of vegetation is typically calculated by measuring tree canopy density, while the understory vegetation is typically not included [19,35]. We know little about the impact of understory vegetation on human health. In a study about natural environments and birth outcomes, results suggest increased vegetation height is predictive of small for gestational age births, but that the greatest predictive power came from landscapes with both high vegetation and also variation in vegetation height. In contrast, homogenous landscapes such as grass fields or dense forests were not predictive [41]. In another study examining greenspace exposure and academic performance, the amount of turfgrass cover showed scattered negative associations with test scores [42].

In addition to the types of vegetation being examined, how we measure the density of vegetation may also matter. As all of us can testify, top-down views of a setting are quite different from eye-level views of the same place. Vegetation density measures are also different from these two perspectives [16,19]. Researchers have used multiple methods to improve their estimations of greenness at eye-level. In some studies, researchers used eye-level pictures or made videos in various categories (such as nature, city) to mimic the way people perceive the environment [43,44]. In experimental settings, researchers have taken pictures in similar areas where people live and produce 3D videos to mimic people’s daily exposure to nature [19]. Another alternative is to expose participants to the real environment. Participants in one study were required to walk or finish tasks in either natural or urban settings as a way to test the impacts of different environments on health [1,23,45].

In addition, people’s personal daily environments can be quite different from the pictures they view in an experiment or the places where an experiment is conducted. Researchers have been searching for alternatives to measure exposure to nature in daily life. Global Position System (GPS) devices have been used to track people’s daily routes and thus to create a more reliable measure of the actual exposure to nature that they have [46,47]. This method is accurate and inclusive for both temporal and contextual variations. Another alternative measurement technique uses Google Street View (GSV) images. Vegetation density in GSV images may be a reliable representation of people’s daily nature exposure, and an increasing number of studies have used it [48,49]. GSV images have been tested as a better way to estimate vegetation density than the top-down perspective method especially for vertical greenness and dense urban areas [9].

In this study, we evaluate the relationship between people’s daily variation in exposure to vegetation and health outcomes by quantifying people’s exposure to nature in different vegetation categories (tree canopy and understory vegetation), and from different perspectives (top-down vs. street view). For the top-down vegetation measurement, we differentiated the tree canopy cover and the understory vegetation and also incorporated a measure of the time spent exposed to the nearby vegetation. In addition to measuring the density of vegetation around people’s homes and places where they work and often visit, we have also measured exposure to vegetation when they are travelling to those places by using Google Street View images.

We address the following questions:

(1) To what extent are variations in daily exposure to vegetation density (e.g., in neighborhoods, daily destinations, and routes) associated with human health? That is, do higher levels of daily exposure to nature predict better health outcomes?
(2) To what extent are variations in exposure to different types of urban nature (e.g., trees, understory vegetation) associated with human health?

## 4. Methods

### 4.1. Data Collection

In order to understand the relationship of variations in daily exposure to nature on health outcomes, we conducted an online survey in the United States from September to November 2017. The survey was available on Qualtrics for participants 18 years old or older. We posted the survey link on Amazon Mechanical Turk to boost the number of participants. People who finished the entire survey received $5 in their Amazon account. A total of 212 individuals started the survey (91 males and 121 females), and 201 individuals (88 males and 113 females) completed the survey.

### 4.2. Measuring Vegetation Concentration at Eye-Level along Daily Routes

**Figure 1:**
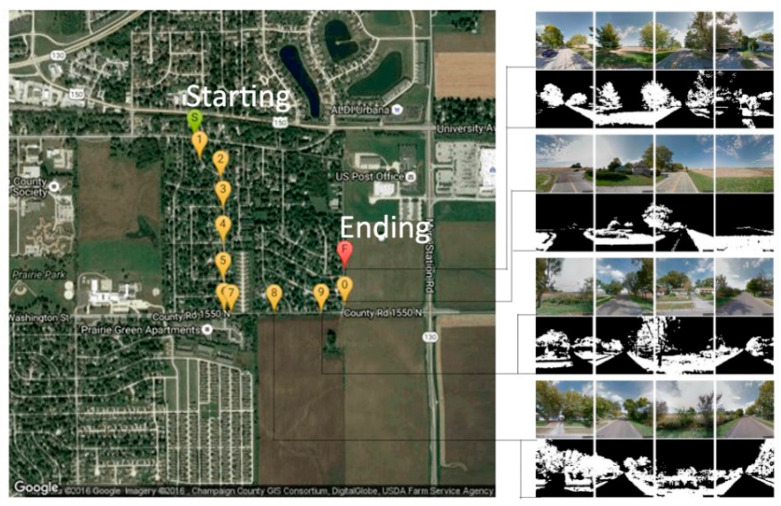
Green Index on A Route: S is the starting point and F is the ending point of the route. We extracted 4 street view images every 20 m along the route. For each image, the percentage of green pixels is calculated which is the vegetation density.

To measure vegetation concentration, we used street view images extracted by Google Map along participants’ daily routes. Each participant reported the three most frequently used routes they took in a typical day. Street view images were downloaded along the routes every 20 m. We downloaded 4 images—facing north, east, south, and west—every 20-m along the route (Figure 1).

The majority of Google Street View images were taken between April and October when leaves and grass are green. Some of the images from California and Texas were taken in November. Due to the warm climate in those areas, vegetation was still green in the images taken in November. We used a tool developed by the National Center for Supercomputing Applications (NCSA) at the University of Illinois at Urbana-Champaign to calculate the percentage of greenness in every image [50].

The average vegetation density for all street view images was generated for all routes, which we defined as the Green Index for each individual. Then we used the length of every route as a weight to calculate the greenness exposure of every participant. The estimation of the Weighted Green Index for each participant is explained in the Formula 1 below:

(1) $$\mathrm{WeightedGreenIndex}=\frac{\mathrm{Length}1*\mathrm{GIRoute}1+\mathrm{Length}2*\mathrm{GIRoute}2+\mathrm{Length}3*\mathrm{GIRoute}3}{\mathrm{Length}1+\mathrm{Length}2+\mathrm{Length}3}$$

where Green Index is the participant’s daily greenness exposure, Length is the distance between the starting and ending point for every route of the participant, and GIRoute is calculated as the average greenness percentage in all the street view images along the route. We also considered the impact of transport mode along participants’ daily routes, classified as either fast (car, bus) or slow (walking, biking). After measuring the Green Index and route length, we calculated the Weighted Green Index for all 201 participants, which ranged from 1.5% to 34.7%.

### 4.3. Measuring Concentration of Vegetation Using Satellite Imagery

We used the National Land Cover Database (NLCD 2011) [34] to estimate land cover characteristics of participants’ homes from the top-down perspective. The database was derived from Landsat 5 Thematic Mapper (TM) satellite images at a 30-m spatial resolution. For the purposes of this study, we included two categories: tree canopy and lower-level vegetation. The tree canopy category consists of areas dominated by trees greater than 5 m tall. The lower-level vegetation category includes areas dominated by shrubs, grass and pasture.

**Figure 2:**
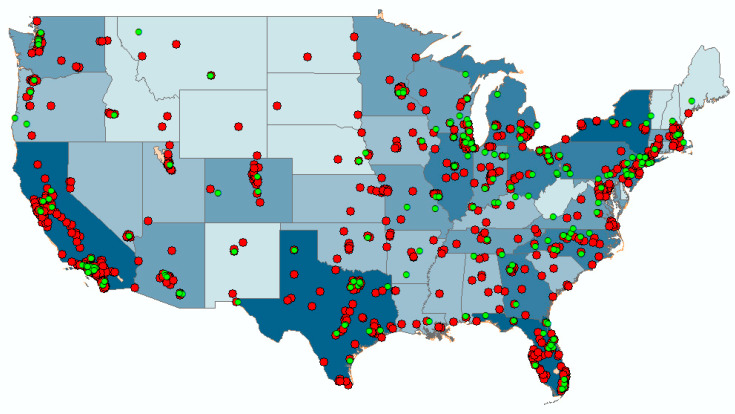
Locations of Home Addresses and City Population: green points represent participants’ home addresses and red points represent cities with a population more than 50,000 people. The darker the color the state is, the higher the population density is that each state.

In the survey, participants indicated their home address and the top three places they most frequently visited. Participants were also asked how many years they have lived at their home address, and how many hours they typically spend at each destination. We geocoded participants’ addresses and destinations and created buffers with radii at 400 m, 800 m, and 1600 m around those locations using the statistical software R. The selection of these particular radii was based on previous studies [4,14,51] in which smaller radii (less than 2 miles) had stronger associations with beneficial outcomes. Because the resolution of NLCD is 30 m by 30 m, we did not use the very small radius (25 m) even though a small radius has been shown to yield significant relationships in previous studies [14,52]. As shown in Figure 2, our participants generally lived in cities with a population of more than 50,000 (estimated in 2016). Thus, we believe the physical environments participants had access to were primarily suburban or urban. In each buffer, we calculate the tree canopy density and the lower-level vegetation density.

To incorporate the temporal factor into the estimation of vegetation concentration, we combined tree density around home and years living at that home into a variable we call Home Tree Years. For lower-level vegetation around the home combined with years living at that home, we created the variable Home Understory Years. Next, we created Destination Tree Duration, the sum of tree density in the area around the destination multiplied by the number of hours typically spent each day at the destination, and Destination Understory Duration, the sum of lower-level vegetation density in the area around the destination multiplied by the number of hours typically spent at the destination.

In sum, we use four independent variables that represent people’s combined physical and temporal exposure to nature: Home Tree Years, Home Understory Years, Destination Tree Duration, and Destination Understory Duration. Each of these variables has three variations with radii at 400 m, 800 m, 1600 m.

### 4.4. Scoring of Standard Questionnaires

We used two standard questionnaires to estimate participants’ overall health (General Health SF-12) and stress level (Perceived Stress Scale).

#### 4.4.1. Summary of General Health SF-12

SF-12 is a standard questionnaire for general health assessment [53]. It is a short yet valid alternative to the SF-36 for use in surveys of general and specific populations as well as large longitudinal studies of health outcomes. The 12 items are classified into two summary scales—Physical Health Summary (PHS) and Mental Health Summary (MHS), which are shown in Appendix A.

The SF-12 Physical Health Score (PHS) and Mental Health Score (MHS) are scored using norm-based methods. The regression weights and constants for both PHS and MHS come from the general US population [54]. The advantages of the standardization and norm-based scoring methods are that results for one participant can be meaningfully compared with another. Their scores can have a direct interpretation in relation to the distribution of scores in the general US population. For example, the average score in the US is 50, so all scores above and below 50 are above and below the average. We combined the individual SF-12 scores to generate the Overall Health Score. The descriptive statistics of the three health scores are in the Table 1 below.

#### 4.4.2. Factor Analysis of Perceived Stress Scale

We used the Perceived Stress Scale (PSS) to assess participants’ stress levels [55]. In the Perceived Stress Scale survey, there are 10 questions. We employed factor analysis with SPSS Statistics 25 to analyze components that underlie the 10 questions. Like other surveys in social science, questions in PSS are often correlated and not independent. Thus, we chose the oblique rotation method in Factor Analysis. The factor loading cutoff was 0.4. Results indicate that the 10 questions in the Perceived Stress Scale grouped into two categories (Appendix B). The first component represents the capability to cope with stress, which we labeled Stress Resilience. The second component represents the ability to have things under control, which we labeled Under Control. After factor analysis, we calculated the weighted average for both components and created a summary score: Overall Stress.

## 5. Results

Results are presented in four sections. First, we examine the extent to which demographic characteristics are related to health outcomes. Second, we evaluate nature exposure around participants’ home address and assess its relationship to health outcomes. Third, we examine the relationship between exposure to nature around participants’ three most frequent destinations and health outcomes. In the final section, we use route greenness to represent people’s daily nature exposure and test its relationship with health outcomes.

For our health analyses, we examine six health indicators: Physical Health, Mental Health, Overall Health, Stress Resilience, Under Control, and Overall Stress. The six health scores do not normally distribute and therefore they violate assumptions of regression. Thus, we use the two-step approach to transform the scores so they fit a normal distribution [56]. The transformed data prove to be normally distributed, and the homogeneity of variance holds.

### 5.1. Demographic Characteristics and Health

We asked five questions related to participants’ gender, age, ethnicity, education and income. 85% of the participants are white and 42% of them have at least a bachelor’s degree (Appendix C). Economic status and social status have been shown to impact human health [57]. Thus, we built a multiple regression model to explore the relationship between the demographic characteristics and health outcomes (Table 2 and Table 3). Among the five characteristics, only income is associated with mental or physical health and the other four demographic variables are not significantly associated with health outcomes. Income is a significant predictor of Mental Health (*p* < 0.05), Overall Health (*p* < 0.05), and Under Control (*p* < 0.01). Age is a significant predictor of Under Control (*p* < 0.05) Thus, all regressions that follow include income and age prior to entering any other independent variable examined below.

### 5.2. Does Exposure to Nature around Home Predict Health?

To what extent does the combination of years living in a home and the density of trees near one’s home (Home Tree Years) predict health? To answer this question, we used Home Tree Years with various radii along with income and age as independent variables and the six health scores (Physical Health, Mental Health, Overall Health, Stress Resilience, Under Control, and Overall Stress) as the dependent variables.

Table 4 describes the relationship between these variables. Notice that most significant relationships for trees are positive, indicating that the greater the exposure to trees around home is associated with better participants’ health. Individuals living in neighborhoods with higher levels of exposure to trees have better Overall Health and report feeling more Under Control. The significant results are between Home Tree Years at 800 m (*p* < 0.05) and Overall Health; Home Tree Years at 400 m (*p* < 0.05), 800 m (*p* < 0.05) and 1600 m (*p* < 0.05) and Under Control. We have also found marginally significant results for Physical Health and Stress Resilience. The relationships between Home Tree Years at 800 m (*p* < 0.1) and 1600 m (*p* < 0.1) and Physical Health are positive; but Home Tree Years at 400 m (*p* < 0.1) is negatively associated with Stress Resilience. The results of Home Tree Years and stress resilience are only marginally significant at the radius of 400 m.

To what extent does the combination of years living in a home and the density of trees near one’s home (Home Understory Years) predict health? To answer this question, we took the Home Understory Years with various radii along with income and age as the independent variable and the six health scores (Physical Health, Mental Health and Overall Health, Stress Resilience, Under Control, Overall Stress) as the dependent variables.

Unlike the tree canopy results, the relationships for understory vegetation are significant and negative, which hints at the possibility that the greater the density of understory plants around the home the worse an individual’s mental health status will be. The significant results are between Home Understory Years at 400 m (*p* < 0.05) and Stress Resilience; and Home Understory Years at 800 m (*p* < 0.05) and 1600 m (*p* < 0.05) and Overall Stress (Table 4). However, the relationships between Home Understory Years at 1600 m and Physical Health are positive (*p* < 0.05).

### 5.3. Does Exposure to Nature around Destinations Predict Health?

To what extent does combination of tree density around one’s destinations and the time spent at those destinations (Destination Tree Duration) predict our measures of health? To answer this question, we used Destination Tree Duration with various radii along with income and age as independent variables, and the six Health indicators (Physical Health, Mental Health, Overall Health, Stressed Resilience, Under Control and Overall Stress) as dependent variables.

We wondered whether Destination Tree Duration predicts both General Health and Stress but found no relationships. That is, the density of trees around participants’ destinations and the time they spent at those destinations was not related to any of the health outcomes (Table 5).

To what extent does the density of understory vegetation and time spent at multiple destinations (Destination Understory Duration) predict health? To answer this question, we used Destination Understory Duration with various radii along with income and age as independent variables, and the six Health indicators as dependent variables.

All of the relationships are negative, which indicates that higher density of understory vegetation around destinations is associated with worse mental health status. The significant results are between Destinations Understory Durations at 400 m (*p* < 0.05), 800 m (*p* < 0.05) and Mental Health; Destinations Understory Durations 400 m (*p* < 0.05), 800 m (*p* < 0.05) and 1600 m (*p* < 0.05) and Under Control; Destinations Understory Durations at 400 m (*p* < 0.05), 800 m (*p* < 0.05) and 1600 m (*p* < 0.05) and Overall Stress (Table 5).

### 5.4. Route Greenness and Health

To what extent does the density of vegetation along the routes one takes to regular destinations predict health outcomes above and beyond the demographic characteristics? In the following analysis, we explore the relationship between Weighted Green Index and the six health outcomes in multiple regression models. Variables of income, age and Weighted Green Index are independent variables, which estimate the contribution of the Weighted Green Index to each one of the six health indicators—Overall Health, Physical Health, Mental Health, Overall Stress, Stress Resilience, and Under Control. Our hypothesis is that, after controlling for demographic information, the Weighted Green Index will be a significant predictor of the health outcomes.

Notice that, in Table 6, for people with the same demographic characteristics, the greater the Weighted Green Index is, the more likely a participant is to report that they have things under control. We have also taken transport modes—fast and slow—into account. For people who used slow transport modes (walking and biking), the Weighted Green Index is marginally, positively significant with higher overall stress.

## 6. Discussion

The survey of 201 participants from the United States demonstrates that greater daily exposure to trees is associated with better health outcomes. Specifically, higher neighborhood concentrations of tree canopy were related to better physical health and overall health. Compared to people who live with less tree canopy nearby, those who live in neighborhoods with more tree cover reported a greater capacity to have things under control, better physical health, and better overall health. Understory vegetation, however, was negatively associated with mental health. People who live in, or work in, areas with higher densities of understory vegetation reported lower levels of mental health and less capacity to cope with stress. Still, we found that greater exposure to understory vegetation around a person’s home was associated with better physical health.

In addition, we found that people who took greener routes to their daily destinations reported they had a greater capacity to have things under control than individuals who took routes with less vegetation. Compared to the time people spent indoors, the time spent commuting was relatively limited, but even so, the density of vegetation along travel routes was significantly and positively associated with reports that people had things under control. In the paragraphs that follow, we consider the contributions of these findings, their implications, and the questions they raise for future research.

### 6.1. Contributions

In this study, we explored the relationship between daily exposure to nature and people’s health outcomes. In North American today, the majority of people spend most of their time indoors, either at work or at home (American Time Use Survey Summary, 2019). Therefore, we calculated the vegetation densities within different buffer distances (400 m, 800 m, and 1600 m) around people’s homes and three of their daily destinations (e.g., work places, athletic facilities, coffee shops). Previous studies have primarily focused on the concentration of nature within specific buffer zones [4,14], but few studies have explored the extent to which time spent in these locations (in hours or years) is related to health outcomes. A number of scholars have suggested that the temporal dimension should be included in our measure of exposure to nature [7,52,58]. In this study, we found that the amount of time spent in places did play a role in the relationship between vegetation density and some health outcomes. Still, our findings come from a cross-sectional study. The results can only show the combined effects of time and vegetation density. Further research with longitudinal data is needed to strengthen our understanding of the extent to which time in a place interacts with the density of vegetation in that place.

One thing to note here is that the independent variable we created to represent exposure to nature around places of interest combines both vegetation density and time, which means that the larger the value is, the greater the density or the longer the time. The longer people live in a neighborhood or stay in a particular place, the stronger the associations between physical settings and health outcomes. For individuals who lived similar lengths of time at their address, the greener the neighborhood, the better the health outcomes. For individuals who lived in neighborhoods with similar levels of tree canopy, the longer they live there, the better they reported their health to be. Our findings demonstrate that time has an impact on the relationship between vegetation density and health outcomes. It is interesting to note that when we included people’s exposure to nature as a variable in multiple regressions without also including time as a factor, the regressions did not predict health outcomes. The interaction between nature density and time is an important finding from this work.

Our study supports previous research showing that higher densities of trees is associated with human health [9,13,19,32], but the findings that understory vegetation has a small but significant negative association with health is new and important. The discrepancy between trees and understory vegetation suggests that people may perceive these types of nature differently. Some of the mechanisms underlying the relationship between exposure to trees and health are well established. Exposure to trees provides opportunities for people to relax and take a break from demanding tasks, which eventually contribute to both stress reduction and attention restoration. We have less understanding, however, of the health benefits of understory vegetation. Previous studies suggest that people do not perceive understory vegetation as a resource that promotes health [59]. A recent study found that local residents were unwilling to pay a small fee to support the maintenance of understory green infrastructure—this was especially the case when residents did not realize the potential benefits of such plantings [60]. Preference studies also suggest many people do not like landscapes they classify as messy. Some understory vegetation can appear messy [61,62]. Even though landscape architects and designers promote understory plantings as critical components of sustainable landscapes, lay people sometimes object to these plantings because of the possibility they pose to attract pests and litter [60].

In this study, we found negative relationships between the amount of understory vegetation and stress, both at home and at daily destinations. Greater concentration of understory vegetation around home was associated with poorer stress status and worse mental health. We obtained similar results from the understory vegetation density at daily destinations, which were negatively associated with the capacity to have things under control, overall stress, and mental health. On the contrary, however, we found the relationships between exposure to understory vegetation around home was positively related to physical health. This finding suggests that we need a finer classification of understory vegetation. These inconsistent results suggest we need a better understanding of the extent to which understory vegetation (e.g., species selections, planting design, levels of maintenance) impacts a variety of measures of health and wellbeing. According to the NLCD Legend for Land Cover Class Descriptions, understory vegetation includes categories such as scrubland, grassland, sedge and pastureland. Given the rather coarse 30 m by 30 m resolution of the NLCD data, we do not have fine-grained details about the characteristics of the understory vegetation in this study other than to know that to be classified as understory vegetation, there was no tree canopy present in the 30 m by 30 m square. Finer grained details that allow us to make richer measures of understory vegetation are necessary if we are to better understand of the relationship between understory vegetation and health outcomes.

Furthermore, it is possible that our classification of the tree category included some understory vegetation. In this study, we did not examine the impacts of possible combinations of trees and understory vegetation on health. Previous research has found that the combination of trees and understory vegetation may reduce the risk of poor birth outcomes for human infants [41]. The findings regarding understory vegetation in this paper are consistent with a study of birth outcomes in that researchers suggest it may be best to combine understory vegetation (e.g., grass) with trees. In most cases, in public and private settings, combining these two planting types can happen. In rare instances, the combination of trees and understory vegetation may not be possible (e.g., on some rooftop gardens it may not be possible to plant trees). Designers and landscape managers need to understand that large swaths of understory vegetation that lack trees might have negative consequences for health and wellbeing.

Finally, we measured greenness along commuting routes using Google Street images. The time our participants spent along these routes was small compared to the time people stayed at home or at work. Thus, one might imagine that the density of vegetation along the routes has little impact on measures of health. The findings, however, show that the greener the routes, the more likely people were to report that they had things under control. The Weighted Green Index, which measured both the density of vegetation and the time spent exposed to that vegetation while on commuting routes, provides a closer look at the vegetation density available at eye-level. This measure is an alternative to typical measures of nature exposure in that it includes time of exposure. The Weighted Green Index reflects the vegetation density dynamic in nature exposure when people travel on their daily routes. Using the street view images allowed us to differentiate the most accessible nature from that which is available on maps but could not be seen on the streets when people were travelling along their routes.

We have employed vegetation densities in buffers around participants’ homes and daily destination, as well as along commuting routes at eye-level. It is important to consider the correlation among these variables. The places for buffers and daily routes are different, so we did not examine the correlation between green index and trees/understory around homes and at destinations. However, the calculation of tree canopy and understory vegetation used data in the same places, so we also examined their correlation. Results show that tree canopy density and understory vegetation in the same buffer are not significantly correlated.

### 6.2. Implications

We found that higher levels of tree canopy concentrations in people’s neighborhoods and at their daily destinations was associated with individuals reporting that they had things under control, experience less stress, and that they had better physical, mental, and overall health. At this point, it is not surprising that trees can promote health [63]. The more important issue concerns where trees are most needed. This is especially important for marginalized or disadvantaged individuals who have few resources to escape to more natural settings. People with greater amounts to money and time are more likely to have access to natural landscapes other than trees on their street. Compared to other investments designed to promote public health, planting trees in neighborhoods that have few trees may have both relatively lower costs and perhaps higher impact. After installing green infrastructure in Philadelphia, for instance, researchers found consistent and statistically significant reductions in narcotics possession near treatment sites [64]. In many cities, vacant land is a significant economic problem that impacts residents’ health and safety [65]. Researchers found that nature-based interventions, such as turning vacant lots into small green spaces, can improve health and safety for residents of urban environments [66]. Greening initiatives can also bring economic benefits via the addition of construction and maintenance jobs in local communities and neighborhoods, but more research on the return-on-investment is needed [66].

Our finding that the amount of time spent in landscapes is a predictor of health outcomes suggests we should encourage people to seek out green neighborhoods, schools, and workplaces. It also suggests that we should plant more trees in places that have little vegetation. City administrators and designers should take advantage of tree canopy as a way to promote public health and alleviate inequality.

Our final implication concerns understory vegetation. We hope the findings here do not discourage designers from including understory plants in the places they create. Instead of avoiding understory plantings, landscape architects should be sure to combine them with trees.

### 6.3. Future Research

In this study, participants were required to identify the top three destinations they typically go to on a daily basis and were asked to identify the landmarks along the way to help us locate their routes. Some of these commutes were several miles and it is possible that, at times, individuals varied the routes they took to these destinations. Even though we have asked people for the transport mode for each route, people may also have occasionally changed the mode used. These inconsistencies prevent us from developing a more accurate commuting map. In the future, researchers might use apps or GPS devices to create finer-grained measures of the routes people take to gain a better understanding of people’s exposure to nature. Speed and transportation methods should also be considered.

Even though the beneficial impacts of tree canopy on health is well established, future research can explore other neighborhood aspects to extend our knowledge. There are emerging hints that biodiversity may have a positive relationship to human health. Future research may explore levels of diversity associated with various levels of tree canopy density and the effects on human health.

The temporal dimension also needs further research. Longitudinal data needs to be collected regarding the extent to which the nature-time exposure interaction impacts health. For those who have lived at their current address for less than three years, researchers should investigate their exposure to nature at their previous addresses.

We need to understand more about the relationship between understory vegetation and human health outcomes. This is critical because trends in landscape architecture and urban design increasingly employ tree and understory plantings. The inconsistent findings described above beg for future research that employs a finer grain classification of understory vegetation. We need to better understand the extent to which understory vegetation (e.g., species selections, planting design) impacts health and wellbeing. Given the 30 m by 30 m resolution of our data, we do not have fine-grained details about the characteristics of the understory vegetation in this study other than to know that it did not include tree canopy. In order to better understand the relationships between understory vegetation and health outcomes, we need to measure more details about the understory vegetation types, the extent of messiness, and the plant diversity. These variables should be measured in future studies.

In a study of urban green infrastructure, messy-looking bioretentions were less preferred by lay people than by designers [15]. We know from previous research that messiness in plantings is a strong and negative predictor of the extent to which people like or prefer planting designs. Future research might explore the extent to which messiness in plantings impact human health outcomes. To the extent that species diversity in understory plantings impacts perceptions of messiness, it seems useful to explore the relationship between the diversity of understory vegetation and human health outcomes.

The findings of understory vegetation provide empirical evidence for an overlooked category of nature. These results suggest that higher concentrations of lower level vegetation are associated with negative health outcomes. However, understory vegetation is a very broad concept which includes many species and combinations. Future studies can explore different types and combinations of vegetation, such as trees with understory vegetation vs. only trees, and understory vegetation in various species vs. only one species. Understanding the various species and complex combination of nature forms can provide valuable suggestions for community design and urban planning.

Finally, participants in this study were not representative of the US population as a whole. According to the US Census (2019), 60.4% of the population is white and 31.5% of people hold a bachelor degree or higher. In our sample, 85% of the participants were white and 42% of them held at least a bachelor’s degree (Appendix C). The participants in our study are skewed toward white people who have received higher education. In future studies, scholars should seek a more representative sample of the US population or perhaps even over-sample minority populations so that we can gain a better understanding of the extent to which exposure to various forms of green infrastructure as associated with health outcomes.

## 7. Conclusions

In this study, we have developed a more comprehensive measure of people’s daily exposure to nature, and found that combined vegetation density and time spent near nature is significantly associated with health outcomes. The results add evidence to our knowledge that higher density of trees is associated with both physical and mental health. We found that people benefit from having higher densities of trees around their homes and along their daily routes.

We also found that higher levels of understory vegetation were associated with better physical health but worse mental health and more stress. As understory vegetation is an important form of urban nature in stormwater management and urban design, it is not possible to exclude this entire type of nature in our cities. Therefore, our challenge is to incorporate various forms of nature in cities in ways that promote health and wellbeing. One way to do so appears to be that we should plant trees along with understory vegetation.

We used Google Street View images to estimate people’s exposure to nature along their daily routes. The results support our hypothesis that higher densities of vegetation are associated with better health outcomes. The process of using Google Street View images was not too labor intensive and suggests a useful strategy for measuring exposure to nature.

This study provides us a new perspective regarding the relationships between nature exposure and health outcomes. The results can help urban designers and administrators make healthier cities.

## Figures and Tables

**Table 1:** Summary of Physical, Mental and Overall Health Measures.

	Minimum	Maximum	Mean	Std. Deviation
Physical Health Score (PHS)	26.15	65.44	46.68	7.67
Mental Health Score (MHS)	14.57	60.76	43.43	9.81
Overall Health Score	51.24	117.33	90.12	13.84

**Table 2:** Multiple Regressions between Five Demographic Variables and Health Outcomes of SF-12.

Demographic Information	Overall Health		Physical Health		Mental Health	
	Unstandardized Coefficient	Standard Error	Correlation Coefficient	Standard Error	Correlation Coefficient	Standard Error
Age	0.86	1.29	−0.26	0.71	1.73	0.91
Income	0.78 *	0.31	0.27	0.17	0.46 *	0.22
Gender	1.36	1.90	0.14	1.05	1.58	1.35
Ethnicity	1.12	0.93	0.70	0.51	0.32	0.66
Education	0.35	0.71	−0.07	0.39	0.18	0.51

*, *p* < 0.05.

**Table 3:** Multiple Regressions between Five Demographic Variables and Health Outcomes of Perceived Stress Scale (PSS).

Demographic Information	Overall Stress		Stress Resilience		Under Control	
	Correlation Coefficient	Standard Error	Correlation Coefficient	Standard Error	Correlation Coefficient	Standard Error
Age	1.01	0.54	0.48	0.45	0.71 *	0.32
Income	0.16	0.13	−0.04	0.11	0.24 **	0.08
Gender	0.72	0.80	0.90	0.65	0.19	0.47
Ethnicity	−0.02	0.40	0.19	0.32	−0.27	0.23
Education	0.08	0.30	−0.05	0.24	0.24	0.17

*, *p* < 0.05, **, *p* < 0.01.

**Table 4:** Relationship between Nature Exposure around Home and Health Outcomes.

	SF-12						Perceived Stress Scale					
Home Year Vegetation	Overall Health		Physical Health		Mental Health		Overall Stress		Stress Resilience		Under Control	
	B	SE	B	SE	B	SE	B	SE	B	SE	B	SE
Trees 400 m	2.78 +	1.61	0.78	0.93	1.56	1.14	−0.20	0.71	−0.98 +	0.58	0.93 *	0.40
Trees 800 m	3.56 *	1.52	1.51 +	0.88	1.41	1.08	0.03	0.67	−0.69	0.55	0.84 *	0.38
Trees 1600 m	2.78 +	1.61	1.53 +	0.85	1.19	1.04	0.10	0.65	−0.56	0.53	0.74 *	0.37
Understory 400 m	0.08	3.76	2.64	2.14	−2.93	2.65	−2.98 +	1.63	−2.28 *	1.34	0.12	0.94
Understory 800 m	−0.39	3.80	3.61+	2.15	−3.87	2.68	−3.45 *	1.65	−2.24 +	1.35	−0.45	0.95
Understory 1600 m	0.15	3.42	4.43*	1.92	−3.82	2.40	−3.43 *	1.48	−2.17 +	1.21	−0.61	0.85

+, *p* < 0.1, *, *p* < 0.05.

**Table 5:** Relationship between Nature Exposure around Destinations and Health Outcomes.

Destination Duration Vegetation	Overall Health		Physical Health		Mental Health		Overall Stress		Stress Resilience		Under Control	
	B	SE	B	SE	B	SE	B	SE	B	SE	B	SE
Trees 400	−0.14	0.31	−0.10	0.14	−0.14	0.22	−0.11	0.13	−0.12	0.11	0.06	0.08
Trees 800	0.07	0.24	0.01	0.13	−0.03	0.17	−0.04	0.10	−0.05	0.08	0.05	0.06
Trees 1600	0.07	0.21	0.01	0.12	−0.02	0.15	0.00	0.09	−0.02	0.07	0.05	0.05
Under 400	−0.43	0.32	0.13	0.18	−0.47 *	0.23	−0.36 *	0.14	−0.21 +	0.11	−0.18 *	0.08
Under 800	−0.33	0.31	0.18	0.17	−0.43 *	0.22	−0.30 *	−0.13	−0.17	0.11	−0.16 *	0.07
Under 1600	−0.22	0.27	0.18	0.15	−0.34 +	0.19	−0.36 *	0.14	−0.10	0.09	−0.17 *	0.07

+, *p* < 0.1; *, *p* < 0.05.

**Table 6:** Relationship between Weighted Green Index Along Daily Routes and Health Outcomes.

Green Index (GI) on Routes	Overall Health		Physical Health		Mental Health		Overall Stress		Stress Resilience		Under Control	
	B	SE	B	SE	B	SE	B	SE	B	SE	B	SE
GI	−0.08	0.14	−0.04	0.08	−0.06	0.10	0.03	0.06	−0.04	0.05	0.08 *	0.04
GI Fast	−0.06	0.12	−0.04	0.07	−0.04	0.09	0.00	0.05	−0.03	0.04	0.05	0.03
GI Slow	−0.11	0.13	−0.05	0.07	−0.05	0.09	0.10 +	0.06	0.05	0.05	0.04	0.03

+, *p* < 0.1; *, *p* < 0.05.

## Appendix A

**Table A1:** Physical Health Summary (PHS) and Mental Health Summary (MHS) of General Health SF-12.

SF-12	Questions
Component 1—Physical Health Score (PHS)	In general, would you say your health is: The following items are about activities you might do during a typical day: moderate activities, such as moving a table, pushing a vacuum cleaner, bowling or playing golf. Does your health now limit you in these activities? If so, how much? The following items are about activities you might do during a typical day: climbing several flights of stairs. Does your health now limit you in these activities? If so, how much? During the past 4 weeks, have you had any of the following problems with your work or other regular daily activities as a result of your physical health: accomplished less than you would like? During the past 4 weeks, have you had any of the following problems with your work or other regular daily activities as a result of your physical health: were limited in the kind of work or other activities? During the past 4 weeks, how much did pain interfere with your normal work (including both work outside the home and housework)?
Component 2—Mental Health Score (MHS)	7. During the past 4 weeks, have you had any of the following problems with your work or other regular daily activities as a result of any emotional problems (such as feeling depressed or anxious): accomplished less than you would like? 8. During the past 4 weeks, have you had any of the following problems with your work or other regular daily activities as a result of any emotional problems (such as feeling depressed or anxious): were limited in the kind of work or other activities? 9. These questions are about how you feel and how things have been with you during the past 4 weeks. For each question, please give the one answer that comes closest to the way you have been feeling. How much of the time during the past 4 weeks—Have you felt calm and peaceful? 10. These questions are about how you feel and how things have been with you during the past 4 weeks. For each question, please give the one answer that comes closest to the way you have been feeling. How much of the time during the past 4 weeks—Did you have a lot of energy? 11. These questions are about how you feel and how things have been with you during the past 4 weeks. For each question, please give the one answer that comes closest to the way you have been feeling. How much of the time during the past 4 weeks—Have you felt downhearted and blue? 12. During the past 4 weeks, how much of the time has your physical health or emotional problems interfered with your social activities (like visiting with friends, relatives, etc.)?

## Appendix B

**Table A2:** Components of Perceived Stress Scale.

Perceived Stress Scale	Questions
Stress Resilience	In the last month, how often have you been upset because of something that happened unexpectedly? In the last month, how often have you felt that you were unable to control the important things in your life? In the last month, how often have you felt nervous and “stressed”? In the last month, how often have you been angered because of things that were outside of your control? In the last month, how often have you felt difficulties were piling up so high that you could not overcome them?
Under Control	In the last month, how often have you felt confident about your ability to handle your personal problems? In the last month, how often have you felt that things were going your way? In the last month, how often have you found that you could not cope with all the things that you had to do? In the last month, how often have you been able to control irritations in your life? In the last month, how often have you felt that you were on top of things?

## Appendix C

**Table A3:** Demographic Information Summary.

		Frequency	Percent (%)
Gender	Male	88	43.8
	Female	113	56.2
Age	18–29	33	16.4
	30–45	110	54.7
	46–59	51	25.4
	60–69	7	3.5
Ethnicity	White	170	84.6
	Black or African American	12	6
	Asian	11	5.5
	Native Hawaiian or Pacific Islander	1	0.5
	American India or Alaska Native	1	0.5
	Others	6	3
Education	Less than high school	2	1
	High school graduation	26	12.9
	Some college but no degree	44	21.9
	Associate degree in college	25	12.4
	Bachelor’s degree in college	84	41.8
	Master’s degree	17	8.5
	Doctoral degree	1	0.5
	Professional degree	2	1
Income	Less than $10,000	6	3
	$10,000 to $19,999	13	6.5
	$20,000 to $ 29,999	29	14.4
	$30,000 to $39,999	26	12.9
	$40,000 to $49,999	12	6
	$50,000 to $59,999	25	12.4
	$60,000 to $69,999	26	12.9
	$70,000 to $79,999	19	9.5
	$80,000 to $89,999	9	4.5
	$90,000 to $99,999	9	4.5
	$100,000 to $149,999	20	10
	$150,000 to $199,999	6	3
	$200,000 to $249,999	1	0.5
